# Dual-strand tumor suppressor miR-193b-3p and -5p inhibit malignant phenotypes of lung cancer by suppressing their common targets

**DOI:** 10.1042/BSR20190634

**Published:** 2019-07-12

**Authors:** Kyung Hee Choi, Chang Hoon Shin, Woo Joo Lee, Haein Ji, Hyeon Ho Kim

**Affiliations:** 1 Department of Health Sciences and Technology, Samsung Advanced Institute for Health Sciences and Technology, Sungkyunkwan University, Seoul, Republic of Korea; 2 Institute for Future Medicine, Samsung Medical Center, Seoul, Republic of Korea

**Keywords:** AJUBA, CCND1, HEG1, Lung cancer, Metastasis, microRNA-193b

## Abstract

Emerging studies suggest that microRNAs (miRNAs) play multiple roles in cancer malignancy, including proliferation and acquisition of metastatic potential. Differentially expressed miRNAs responsible for the malignancy of lung cancer were searched by miRNA microarray using a previously established brain metastatic lung cancer model. Twenty-five miRNAs were down-regulated in brain metastatic lung cancer cells. Among those, miR-193b-3p and -5p were chosen for further studies. Their function in metastatic potential and proliferation was examined using Transwell invasion, wound healing, and colony forming assays. The underlying mechanism of tumor-suppressor miR-193b-3p and -5p was explored using reverse transcriptase quantitative polymerase chain reaction (RT-qPCR), Western blot, Argonaute 2-RNA immunoprecipitation (Ago2-RIP), and reporter assays. Both strands of miR-193b were down-regulated in brain metastatic lung cancer cells and in tissues from lung cancer patients. Overexpression of miR-193b-3p and -5p inhibited invasive and migratory activities and diminished clonogenic ability. Conversely, inhibition of miR-193b-3p or -5p increased the metastatic potential and colony forming ability. Cyclin D1 (*CCND1*), Ajuba LIM Protein (*AJUBA*), and heart development protein with EGF like domains 1 (*HEG1*) were identified as common target genes of miR-193b-3p and -5p. A reporter assay and an Ago2-RIP experiment showed that both miRNAs directly bind to the 3′ untranslated region (3′UTR) of the target mRNA. Knockdown of target gene reduced the proliferative and metastatic potential of primary and metastatic lung cancer cells. Our results demonstrate miR-193b is a dual-strand tumor suppressor and a novel therapeutic target for lung cancer.

## Introduction

Despite the remarkable development of lung cancer therapies, lung cancer has been classified as a major cause of death [1]. Its unfavorable clinical outcomes originate from its high invasive and metastatic potential [2,3]. Within a few months of diagnosis, distant metastasis of lung cancer occurs mainly to the brain and bone [4]. The limited efficacy of lung cancer therapy can be largely attributed to two main difficulties. First, lung cancer shows higher aggressiveness than other cancer types do, which makes prognosis very poor [5]. Second, since conventional anticancer drugs are targeted for lung cancer, effective treatment for metastatic lung cancer is limited [6]. For these reasons, the regulatory molecules that control the metastatic process need to be discovered to build the basis for the development of new therapies.

MicroRNAs (miRNAs) are highly conserved small non-coding RNAs ranging from 19 to 25 nucleotides in length [7]. They are known to regulate target gene expression at the RNA level, which is termed as post-transcriptional gene regulation (PTGR). Generally, miRNAs bind to the 3′ untranslated region (3′UTR) of the target mRNA with only partial complementarity and, thus, down-regulate target genes by degrading their mRNA or inhibiting their translation [8,9]. Depending on the function of the target genes, miRNAs are divided into oncogenic and tumor-suppressing miRNAs, which inhibit tumor suppressors and oncogenes, respectively [10,11]. Emerging evidence indicates that miRNAs are closely involved in several cancer development stages, including proliferation, angiogenesis, migration, and invasion [12].

We have investigated the molecular mechanism whereby lung cancer cells acquire malignant characteristics such as metastatic and proliferative potential. Particularly, we examined the effects of miR-193b-3p and -5p, which are down-regulated in metastatic lung cancer cells on malignant phenotypes of lung cancer cells. Both strands of miR-193b suppress the expression of common target genes. Based on our findings, we identified miR-193b as a dual-strand tumor suppressor.

## Materials and methods

### Cell culture and transfection

Brain metastatic lung cancer cells (PC14PE6/LvBr4) were previously established by repeated left-ventricle injection of lung adenocarcinoma PC14PE6 cells [13,14]. Parental PC14PE6, its derivative PC14PE6/LvBr4, and human lung adenocarcinoma A549 cells were cultured in Dulbecco’s modified Eagle’s medium (DMEM; Hyclone) supplemented with 10% (v/v) fetal bovine serum (FBS) and 1% (v/v) antibiotic-antimycotic solution (GIBCO-BRL) at 37°C and 5% (v/v) CO_2_. For knockdown of miR-193b targets, cells were plated at a density of 3.5 × 10^5^ cells/dish. After 24 h, transfected with the indicated small interfering RNAs (siRNAs) or control (CTRL) siRNA using Lipofectamine 2000 (Thermo fisher Scientific, U.S.A.). The Opti-MEM™ (Thermo fisher Scientific, U.S.A.) is used for transfection medium. The 6 h later, the transfection medium was changed to Dulbecco’s modified Eagle’s medium (DMEM; Hyclone) supplemented with 10% (v/v) FBS. Control siRNA (UUCUCCGAACGUGUCACGU), Ajuba LIM Protein (*AJUBA*) siRNA (GUGUCAAUGGCUCUGUGUA), Cyclin D1 (*CCND1*) siRNA (GCACAUCUUGGCUAUGUAA), heart development protein with EGF like domains 1 (*HEG1*) siRNA (UCGAGAAGAAACAGUAGAGUAAC) and matrix metalloproteinase 16 (*MMP16*) siRNA (CGUGAUGUGGAUAUAACCA) were synthesized by Bioneer (Korea). The overexpression plasmid pcDNA3.2/V5 hsa-mir-193b (Plasmid #26318) was purchased from Addgene. Synthetic miRNA mimics (Pre-193b-3p, Pre-193b-5p) and miRNA antisense inhibitors (Anti-193b-3p and Anti-193b-5p) were purchased from Ambion (U.S.A.). To verify miRNA overexpression, the levels of each miRNA in three independent sets were assessed by RT-qPCR using specific TaqMan primers (Supplementary Table S1).

### Western blot and reverse transcriptase quantitative polymerase chain reaction analyses

Cells were washed with ice-cold phosphate-buffered saline (PBS) and lysed using radioimmunoprecipitation (RIPA) buffer containing protease and phosphatase inhibitor cocktail (Roche, Switzerland). Cell lysates were clarified by centrifugation, and the protein concentration was quantified by using Bradford solution (Bio-Rad Laboratories, U.S.A.). Equal amounts of protein were separated by sodium dodecyl sulfate-polyacrylamide gel electrophoresis (SDS/PAGE) and transferred to polyvinylidene fluoride membranes (PVDF; Millipore, U.S.A.). After blocking with 5% bovine serum albumin (BSA) (5 g BSA in 100 ml TBST) or 5% skimmed milk (5 g skimmed milk in 100 ml TBST), membranes were incubated with the indicated primary antibodies overnight. Ajuba (Cell Signaling Technology #4897), cyclin D1 (Cell Signaling Technology #2922), and glyceraldehyde 3-phosphate dehydrogenase (GAPDH; abcam #8245) antibodies were used. After 48-h post-transfection, Total RNA was isolated using TRIzol reagent (Ambion, U.S.A.). Chloroform (Merck, U.S.A.) was added, and the homogenate was allowed to separate into a clear upper aqueous layer, RNA was precipitated from the aqueous layer with isopropanol (Merck, U.S.A.). Isolated total RNA was converted into cDNA using the SuperScript III First-Strand Synthesis System (Invitrogen, U.S.A.). The mRNA was quantified using reverse transcriptase quantitative polymerase chain reaction (RT-qPCR) (ABI PRISM™ 7900HT) and Power SYBR^®^ Green PCR Master Mix (Applied Biosystems, U.S.A.). The following primers were used for amplification: Primary-miR-193b #1 (forward: CGGTTCTCCAAAACTCTTGC, reverse: CCTCCAAAAGCCTCTTTTCC), Primary-miR-193b #2 (forward: AATGGGGACTCACTTCTTGG, reverse: AAACTCATCTCGCCCTCAAA), *AJUBA* (forward: TCCTACAAGCAATGGGGAAG, reverse: AGTCCACTGTGAAGGGGATG), *CCND1* (forward: CCGTCCATGCGGAAGATC, reverse: ATGGCCAGCGGGAAGAC), *HEG1* (forward: TCCTCCAGATGACGGATGTG, reverse: GACGGGTTATACTGTCCGGG), *MMP16* (forward: CACTGGAAGACGGTTGGATT, reverse: TTCCGCAGACTGTAGCACAT) and *GAPDH* (forward: TGCACCACCAACTGCTTAGC, reverse: GGCATGGACTGTGGTCATGAG). The expression levels of miR-193b-3p (MIMAT0002819) and miR-193b-5p (MIMAT0004767) were assessed using specific TaqMan primers (Applied Biosystems, U.S.A.).

### Interaction between miR-193b and target mRNAs

For reporter assay, we constructed dual luciferase vectors (pmirGLO dual-luciferase vector, Promega E1330) containing wild-type (WT) or mutated (MT) miR-193b-3p and miR-193b-5p binding sites (miRNA recognition element, MRE) in the 3′UTR of the target genes. Mutated reporter vectors were constructed by substituting four or five nucleotides in the seed region, abolishing the interaction between miRNA and target mRNAs. Briefly, cells transfected with Con-miR or Pre-miR (Pre-193b-3p or Pre-193b-5p) were seeded into 24-well plates. After 24 h, cells were transfected with either WT or MT luciferase vector. After incubation for 20 h, luciferase expression was measured using a Dual-GLO™ Luciferase Assay System (Promega E2940). In addition to the reporter assay, we assessed the direct interaction between miRNA and target gene mRNA by performing Argonaute 2 (Ago2)-RNA immunoprecipitation (Ago2-RIP). Ago2 antibody (Sigma–Aldrich, U.S.A.) [14] and Dynabeads Protein G (Invitrogen, U.S.A.) were incubated at 4°C with rotation a day prior to the experiment. Cytoplasmic lysates were prepared using PEB buffer containing a protease inhibitor, a phosphatase inhibitor (Roche, Switzerland), and RNaseOUT (Invitrogen, U.S.A.). RNA in Ago2-RIP materials was washed several times with PEB buffer and treated with DNase I (Ambion, U.S.A.) and Proteinase K (Bioneer, Korea). The RNA was isolated with acid phenol (Ambion, U.S.A.) and precipitated with absolute ethanol overnight at −20°C. The enrichment of *AJUBA, CCND1*, and *HEG1* mRNA in Ago2-RIP was assessed by RT-qPCR. Direct binding of miRNAs to the target mRNA was determined by calculating the relative enrichment of target mRNA.

### Determination of proliferative and metastatic potential

To determine clonogenicity, a colony-forming assay was performed. Briefly, transfected cells were plated into six-well plates at a density of 3 × 10^2^ cells/well and cultured in complete medium. After incubation for 2 weeks, cells were fixed with 4% (v/v) paraformaldehyde and stained with 0.2% crystal violet (0.2 g crystal violet in 100 ml distilled water). Clonogenicity was assessed by counting the number of colonies. Metastatic potential was determined by measuring the invasive and migratory abilities of cancer cells. The invasiveness was assessed using BioCoat™ Matrigel^®^ Invasion Chambers (Corning, U.S.A.). 2 h before start cell trypsinization, lower chamber and upper chamber were filled with serum-free medium. Equal numbers of cells in serum-free medium were added to the upper chamber. Cellular invasion was triggered by the addition of complete medium containing FBS to the lower chamber. After 24 h, cells were fixed with methyl alcohol (Daejung chemicals & metals, Korea) and stained with hematoxylin solution (Sigma–Aldrich, U.S.A.) and 0.5% eosin Y (0.5 g eosin Y in 100 ml alcoholic solution) (Polysciences, Inc., U.S.A.) (H&E). Quantification of invasiveness was performed by counting the number of invaded cells from at least ten fields. The migratory activity was assessed by wound closure assay. Transfected cells were seeded into 12-well plates with high density and then a straight line was scratched by white pipette tips. After 24 h, the migrated distance was measured by AxioVision microscope software (ZEISS, Germany) and Image J.

## Results

### Both strands of *miR-193b* are down-regulated in metastatic lung cancer

**Figure 1:**
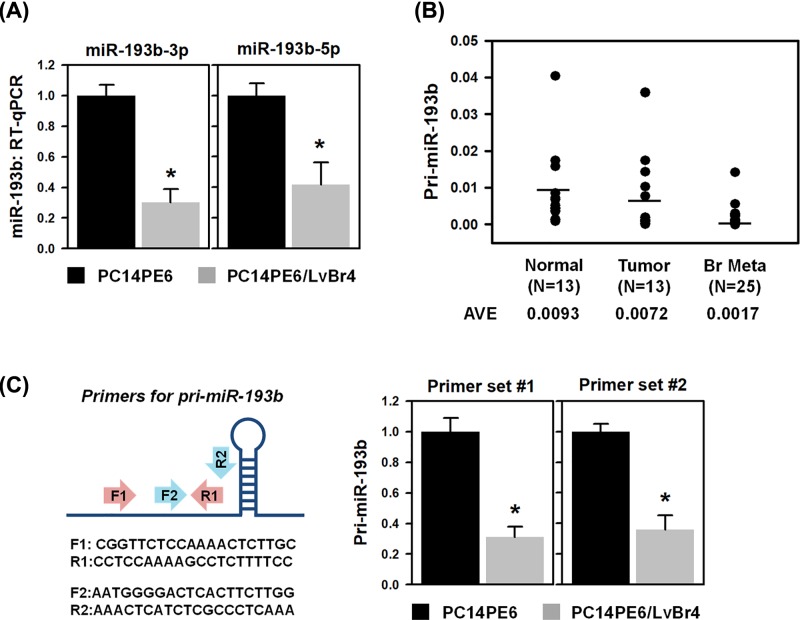
miR-193b-3p and -5p are down-regulated in metastatic lung cancer cells (**A**) To verify down-regulation of miR-193b-3p and -5p, RT-qPCR was performed in three independent replicates using specific TaqMan primers. (**B**) To assess whether miR-193b-3p and -5p were transcriptionally down-regulated, the expression level of pri-miR-193b was determined in three independent replicates using the two different primers shown in the scheme. Results were normalized against *GAPDH* mRNA. In the scheme, F and R refer to the forward primer and reverse primer, respectively. Numbers of primer indicate each set. (**C**) RT-qPCR was performed to detect pri-miR-193b levels in surgical samples from normal lung (N = 13), lung cancer (N = 13), and brain metastatic lung (N = 25). AVE means the average level of primary miR-193b. Data represent mean ± standard deviation (S.D.). Asterisk (*) denotes statistical significance of *P<*0.05 calculated with the Student’s *t*-test.

Previously, we established an *in vitro* model of brain metastatic lung cancer through repeated left ventricle injection and found that brain metastatic lung cancer cells (PC14PE6/LvBr4) had higher metastatic potential, including migratory and invasive abilities, than parental lung cancer cells (PC14PE6) did [15,16]. To search differentially expressed miRNAs between PC14PE6 and PC14PE6/LvBr4 cells, we performed GeneChip miRNA 3.0 microarray (Thermo fisher Scientific, U.S.A.) and identified 25 miRNAs that were down-regulated in PC14PE6/LvBr4 cells (Supplementary Figure S1A). Among them, our research focused on the tumor-suppressing roles of miR-193b because the expression level of both its strands (i.e., 3p and 5p) was considerably decreased in metastatic cells (Supplementary Figure S1B). When two strands generated from one precursor miRNA (pre-miRNA) exhibit the same tumor-suppressing function, such miRNAs are called dual-strand tumor suppressor [17]. For example, miR-145-3p and -5p cooperatively regulate cancer progression and aggressiveness of lung and bladder cancer by suppressing metadherin (MTDH) and ubiquitin-like with PHD and ring finger domains 1 (UHRF1), respectively [17,18]. To confirm that miR-193b-3p and -5p were highly expressed in non-metastatic cells, RT-qPCR was performed using specific primers for miRNAs. As shown in Figure 1A, we observed that miR-193b-3p and -5p were significantly down-regulated in metastatic PC14PE6/LvBr4 cells. Moreover, it was found that the expression of primary miR-193b was also decreased in metastatic cells (Figure 1B), which suggests that the low expression level of miR-193b-3p and -5p was due to the reduced transcription of its primary form. RT-qPCR analyses using tissues from patients with lung cancer and brain metastases revealed that the expression level of primary miR-193b further decreased as the cancer developed into brain metastasis (Figure 1C).

### Ectopic expression of miR-193b-3p and -5p inhibits the metastatic potential and proliferation of lung cancer

**Figure 2:**
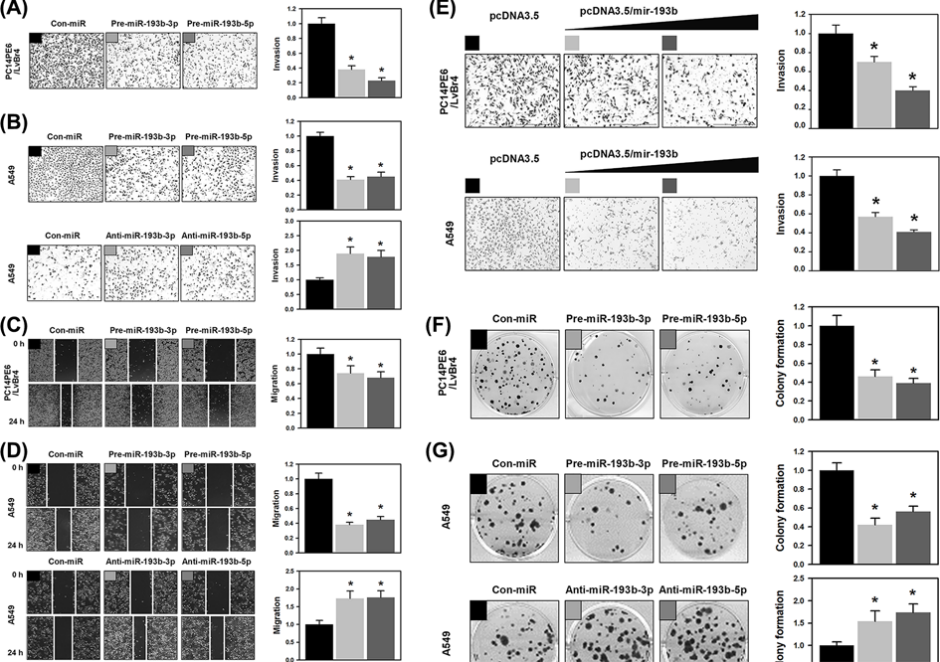
Ectopic expression of miR-193b-3p and -5p inhibits migration, invasion, and proliferation of lung cancer (**A**) For overexpression of miRNA, PC14PE6/LvB4 cells were transfected with precursor-miR-193b-3p and -5p (pre-miR-193b-3p and -5p). Invasiveness was assessed by Transwell invasion assay. (**B**) To examine the effect of miRNA, A549 cells were transfected with precursor-miR-193b-3p and -5p (pre-miR-193b-3p and -5p) or antisense-miR-193b-3p and -5p (anti-miR-193b-3p and -5p) for overexpression or inhibition, respectively. Invasiveness was assessed by Transwell invasion assay. (**C**) PC14PE6/LvBr4 cells were transfected with pre-miR-193b-3p and -5p and migratory ability was determined by wound closure assay. (**D**) After A549 cells were transfected with indicated miRNA mimic or antisense inhibitor, migratory abilities were assessed by wound closure assay. (**E**) To overexpress both strands of miR-193b, PC14PE6/LvBr4 and A549 cells were transfected with pcDNA3.2/V5 hsa-mir-193b (Plasmid #26318). Invasive ability of PC14PE6/LvBr4 and A549 cells was assessed by Transwell invasion assay. (**F**) PC14PE6/LvBr4 cells were transfected with pre-miR-193b-3p and -5p, then proliferative potential was determined by colony forming assay. (**G**) A549 cells were transfected with miRNA mimic or antisense inhibitor for miR-193b-3p and 5p. Colony forming assay was performed to determine proliferative potential of transfected cells. All experiments were performed three times and data represent mean ± S. D. Asterisk (*) denotes statistical significance of *P<*0.05 calculated with the Student’s *t*-test.

Since miR-193b-3p and 5p were down-regulated in brain metastatic PC14PE6/LvBr4 cells, we checked if overexpression of miR-193b-3p or -5p could inhibit the metastatic potential including migratory and invasive abilities. As shown in Figure 2A,C, overexpression of miR-193b-3p or -5p by transfection with each pre-miRNA resulted in the decrease of invasive and migratory abilities of PC14PE6/LvBr4 cells. Since parental PC14PE6 cells are hard to transfect, we tested the effect of miR-193b inhibition on the metastatic potential in A549 lung cancer cells. In accordance with the results obtained with PC14PE6/LvBr4 cells, the invasiveness of A549 cells was considerably diminished by restoring miR-193b-3p or -5p (Figure 2B). In contrast, down-regulation of miR-193b-3p and -5p increased the number of invading cells almost twofold. Next, we assessed the effect of miR-193b-3p and -5p on the migratory ability of A549 cells by wound closure assay (Figure 2D). Ectopic expression of miR-193b-3p or -5p by transfection with each pre-miRNA reduced the migratory potential of A549 cells. On the contrary, decreased expression of miR-193b-3p or -5p through transfection of anti-miRs potentiated the migratory ability of A549 cells. Therefore, overexpression of either 3p or 5p single strand of miR-193b inhibited the metastatic potential. To confirm these results, we tested whether invasiveness was reduced by restoring the level of both strands using the pcDNA/mir-193b plasmid. Overexpression of both miR-193b-3p and -5p dramatically diminished the invasive ability of PC14PE6/LvBr4 and A549 cells (Figure 2E).

To confirm the effect of both miR-193b strands on the proliferative potential, we performed a colony forming assay. The number of colonies was significantly decreased in PC14PE6/LvBr4 cells transfected with miR-193b-3p or -5p precursors (Figure 2F). Similarly, in A549 cells, ectopic expression of miR-193b-3p or -5p inhibited the proliferative potential. Inversely, down-regulation of miR-193b-3p or -5p increased the number of colonies (Figure 2G). Therefore, we concluded that miR-193b-3p and -5p constitute a dual-strand tumor suppressor.

**Figure 3:**
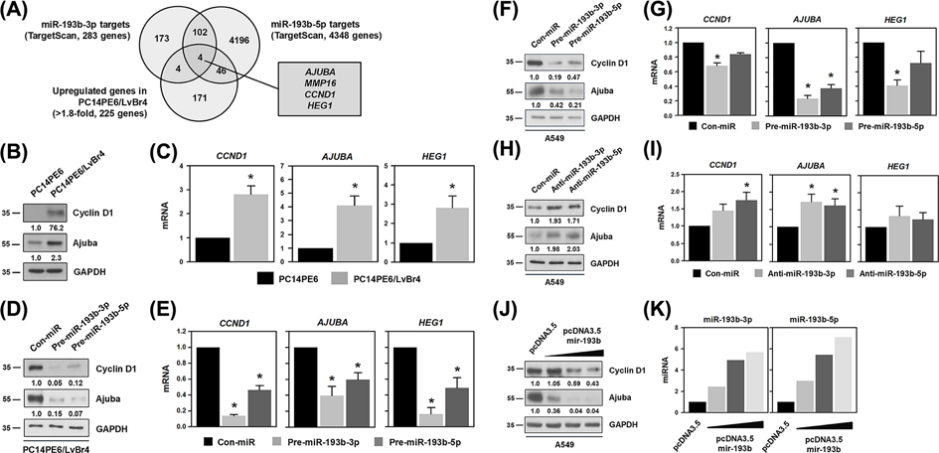
*CCND1, AJUBA*, and *HEG1* are common targets of miR-193b-3p and -5p (**A**) TargetScan algorithm was used to search for common target genes of miR-193b-3p and -5p. Four target genes were selected by comparing the miR-193b common target genes and the list of down-regulated genes in PC14PE6/LvBr4 cells. (**B**,**C**) To compare the expression levels of common target genes (*CCND1, AJUBA*, and *HEG1*) between parental PC14PE6 and brain metastatic PC14PE6/LvBr4 cells, Western blot and RT-qPCR analyses were performed. (**D**–**G**) The effect of miRNA mimics on target gene expression was determined by Western blot and RT-qPCR analyses in PC14PE6/LvBr4 (**D**,**E**) and A549 (**F,G**) cells. (**H**,**I**) To assess the effect of miRNA inhibition, A549 cells were transfected with anti-miR-193b-3p or -5p. The expression levels of target genes were assessed by Western blot and RT-qPCR analyses. (**J,K**) The inhibitory effect of miR-193b on target gene expression was determined by Western blot analysis (**J**). Both strands of miR-193b were efficiently overexpressed upon transfection of A549 cells with pcDNA3.5/mir-193b (**K**). All experiments (except J and K) were performed three times and data represent mean ± S.D. Asterisk (*) denotes statistical significance of *P<*0.05 calculated with the Student’s *t*-test.

To gain further insight in the underlying molecular mechanism of miR-193b-3p and -5p in the malignant processes of lung cancer, we searched for common targets of miR-193b-3p and -5p using the TargetScan prediction algorithm and found that four genes (i.e., CCND1, AJUBA, HEG1, and MMP16) are putative common targets of miR-193b-3p and -5p (Figure 3A). List of other merged genes is shown in Supplementary Figure S2. Although MMP16 was in the list of common target genes and highly expressed in PC14PE6/LvBr4 cells, it was not further studied because (Supplementary Figure S3A–D) (i) the enrichment of MMP16 mRNA in Ago2-RIP was not increased by restoring miR-193b-3p and -5p, and (ii) overexpression of miR-193b-3p or -5p could not suppress the expression of MMP16.

We compared the expression level of common target genes in parental and metastatic lung cancer cells. Since antibodies against HEG1 were unavailable, its expression was assessed only by RT-qPCR. Western blot analysis indicated that metastatic PC14PE6/LvBr4 cells expressed common target genes, including cyclin D1 and Ajuba, at higher levels than parental PC14PE6 cells did (Figure 3B). Since HEG1 antibody was not commercially available, we could not assess the protein level of HEG1. Alternatively we checked the *HEG1* mRNA level by RT-qPCR. Similarly, the mRNA level of common target genes was higher in PC14PE6/LvBr4 cells than in parental PC14PE6 cells (Figure 3C). These results indicated that the levels of miR-193b-3p and -5p are inversely correlated with those of common target genes.

Next, we tested whether miR-193b-3p and -5p suppressed the expression of common target genes. Overexpression of miR-193b-3p or -5p dramatically lowered the protein level of cyclin D1 and Ajuba in PC14PE6/LvBr4 cells (Figure 3D). Moreover, we found that mRNA levels of CCND1, AJUBA, and HEG1 decreased upon overexpression of miR-193b-3p or -5p (Figure 3E). As observed in PC14PE6/LvBr4 cells, protein and mRNA levels of common target genes were diminished by the overexpression of miR-193b-3p or -5p in A549 cells (Figure 3F,G). The effect of miRNA inhibition on the expression of target genes was investigated in A549 cells. Inhibition of miR-193b-3p or -5p using anti-miR increased the protein level of cyclin D1 and Ajuba and their mRNA (Figure 3H). However, HEG1 was not significantly up-regulated by inhibiting either miR-193b-3p or -5p (Figure 3I). To overexpress both strands of miR-193b, A549 cells were transfected with the pcDNA/mir-193b plasmid. As the amount of plasmid increased, the protein levels of cyclin D1 and Ajuba decreased (Figure 3J). The level of miR-193b-3p and -5p was determined by RT-qPCR (Figure 3K).

### miR-193b-3p and -5p directly bind to the 3′UTR of target mRNAs

**Figure 4:**
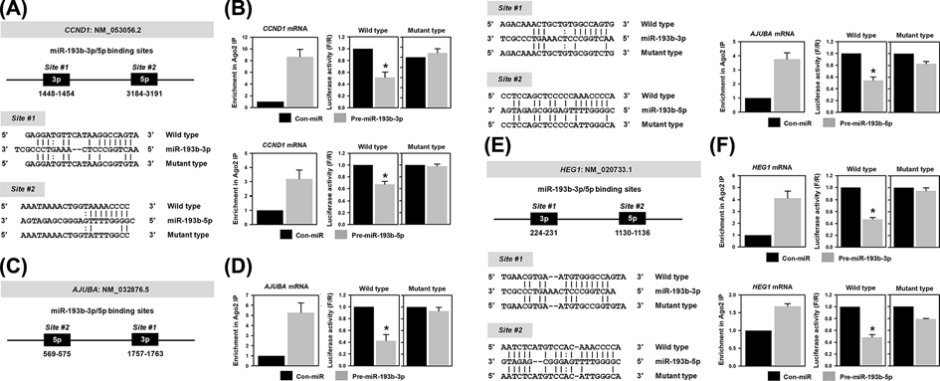
miR-193b-3p and -5p suppress common target genes by directly binding to the 3′UTR of their mRNAs To assess whether down-regulation of common target genes by miR-193b-3p or -5p was due to direct interaction with the 3′UTR of target mRNAs, we constructed a luciferase reporter vector containing wild-type or mutated sequences of the miRNA recognition element (MRE), which are briefly described in the schemes (**A, C** and **E**). To disrupt the interaction between miRNAs and target 3′UTR, four or five nucleotides in the seed region were mutated. Enrichment of target mRNAs was checked by determining their level in miR-193b-loaded Ago2 RISC (left panel of **B,D,F**). The expression of luciferase was assessed by measuring its activity using a Dual-GLO™ Luciferase Assay System (right panel of **B,D,F**). All experiments were performed three times and data represent mean ± S.D. Asterisk (*) denotes statistical significance of *P<*0.05 calculated with the Student’s *t*-test.

We previously selected common target genes (e.g., CCND1, AJUBA, and HEG1) regulated by both strands of miR-193b. To test whether miR-193b-3p and -5p directly bind to the 3′UTR of target mRNAs, Ago2-RIP and luciferase reporter assay were performed. Figure 4 represents the predicted binding sites (WT and mutated sequence) for miR-193-3p and -5p in the 3′UTR of each target mRNAs (Figure 4A,C and E). Compared with control miRNA, all of the common target mRNAs were particularly enriched in Ago2-RIP materials of the sample overexpressing miR-193b-3p (Figure 4B,D and F left panel). Similar to miR-193b-3p, the enrichment of target mRNAs in Ago2-RIP materials increased upon overexpression of miR-193b-5p (Figure 4B,D and F left panel). These results indicate that the RNA-induced silencing complex (RISC) loaded with miR-193b-3p or -5p directly binds to the 3′UTR of target mRNAs, thus suppressing their expression.

In addition to the Ago2-RIP experiment, the direct interaction between miR-193b and target mRNAs was investigated using the luciferase reporter assay. We constructed dual luciferase vectors containing MREs for each miR-193b-3p and -5p in the 3′UTR from target mRNAs including CCND1, AJUBA, and HEG1 (schemes are in Figure 4A,C and E, respectively). To assess the importance of the seed sequence, we also constructed luciferase vectors containing mutated sequences, in which four or five nucleotides were changed in complementary sequences. In all of the vectors harboring WT seed sequence, miR-193b-3p and -5p inhibited the luciferase activity. Conversely, suppression of target genes by *miR-193b-3p* and -5p was abolished by the disruption of the direct binding between miR-193b and the 3′UTR of the target mRNAs by point mutations in the seed sequence. These results demonstrate that miR-193b-3p and -5p inhibit the expression of common target genes (e.g., CCND1, AJUBA, and HEG1) through direct interaction with the 3′UTR of their mRNA.

### Down-regulation of *CCND1, AJUBA*, and *HEG1* is involved for the function of miR-193b

**Figure 5:**
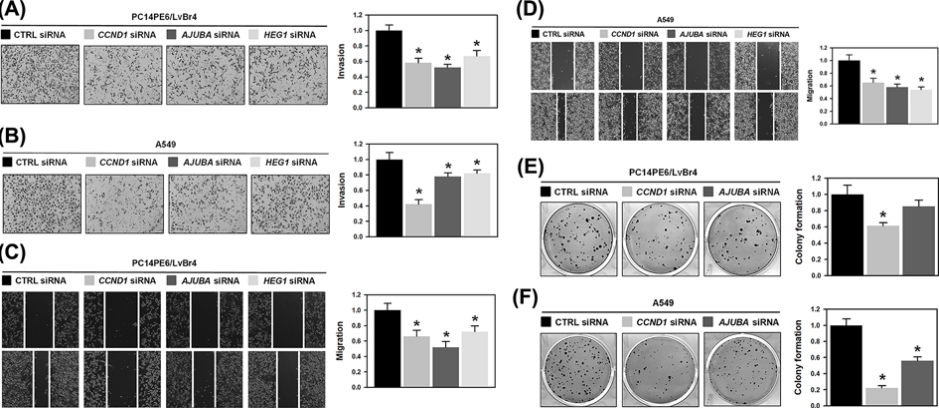
Down-regulation of *CCND1, AJUBA*, and *HEG1* is responsible for the inhibitory effects of *miR-193b-3p* and -5p on lung cancer malignancy PC14PE6/LvBr4 (**A,C**, and **E**) and A549 (**B,D**, and **F**) cells were transfected with specific siRNA targeting *CCND1, AJUBA*, or *HEG1*. Invasive and migratory abilities of transfected cells were tested by Transwell invasion assay and wound closure assay, respectively (A-D). The effect of target gene silencing on the proliferation of lung cancer cells was assessed by colony forming assay (E–F). All experiments were performed three times and data represent mean ± S.D. Asterisk (*) denotes statistical significance of *P<*0.05 calculated with the Student’s *t*-test.

To test whether the down-regulation of common target genes is responsible for miR-193b-elicited inhibition of cancer malignancy, siRNAs targeting CCND1, AJUBA, and HEG1 were prepared. Each siRNAs efficiently decreased the corresponding protein levels (Supplementary Figure S4A–D) and reduced PC14PE6/LvBr4 and A549 invaded cells (Figure 5A,B). Additionally, the migratory ability of those cells was suppressed when the expression of common target genes decreased (Figure 5C,D). Moreover, we checked whether common target genes affected the function of *miR-193b* in the proliferation of lung cancer cells. Knockdown of each target genes inhibited the colony-forming activity of PC14PE6/LvBr4 and A549 cells (Figure 5E,F). Collectively, it was confirmed that the decrease in the expression level of common target genes was closely related to the inhibitory effect of miR-193b on the metastatic and proliferative potential.

**Figure 6:**
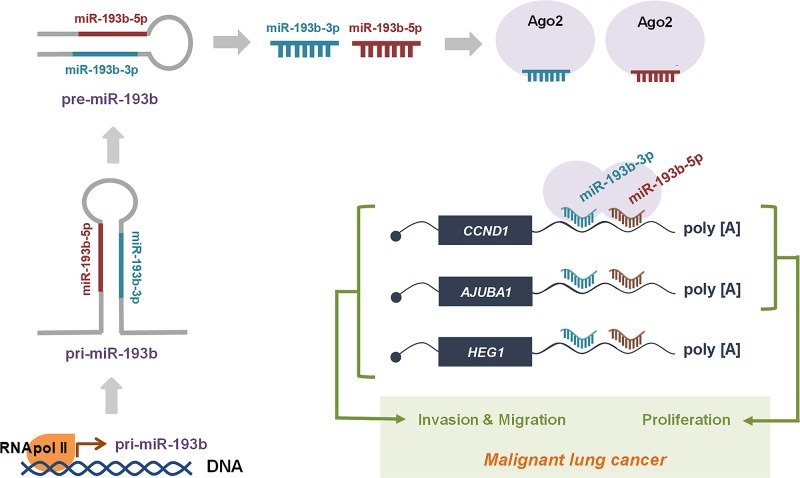
The action mechanism of miR-193b as a dual-strand tumor suppressor is represented in the scheme A detailed description is given in the text.

The action mechanism of miR-193b-3p and -5p consistent with our findings is summarized in Figure 6. Briefly, primary miRNA-193b is transcribed by RNA polymerase II and cleaved to form precursor and mature miRNA by Drosha and Dicer endonucleases, respectively. Both mature miRNAs are loaded into Ago2 and bind to their common target mRNAs such as CCDN1, AJUBA, and HEG1. Through down-regulation of target genes, miR-193b-3p and -5p function as dual-strand tumor suppressor in lung cancer cells.

## Discussion

In general, miRNAs repress the expression of target genes by degrading the mRNA or inhibiting translation in a sequence-dependent manner [19]. Accumulating reports have demonstrated that aberrant expression of miRNAs is closely involved in cancer progression [20]. During the biogenesis of miRNA, two mature miRNAs are generated from one pre-miRNA which is processed from primary miRNA (pri-miRNA) [21]. It is commonly accepted that one strand (major or guide strand) is more abundant and functional than the other strand (minor or passenger strand) [22]. However, several reports suggested that two strands derived from one pre-miRNA exhibit the same tumor suppressing functions [17,18,23]. For example, miR-145-3p and -5p inhibit the aggressiveness of bladder cancer cells by suppressing UHRF1 [17] and function as tumor suppressor in lung squamous cell carcinoma through down-regulation of MTDH [18]. Moreover, miR-139-5p (major) and -3p (minor) are down-regulated in bladder cancer and act as tumor suppressors by targeting matrix metalloproteinase 11 [23]. In the present study, we identified miR-193b-3p and -5p as a new dual-strand tumor suppressor, which inhibits the proliferative and metastatic potential of lung cancer by suppressing common targets, including CCND1, AJUBA, and HEG1.

As a tumor suppressor, miR-193b-3p has been reported to be down-regulated and to inhibit cancer malignancy in various types of cancer. Stathmin 1 (STMN1) was identified as a direct functional target of miR-193b in colorectal cancer [24]. Ectopic expression of miR-193b inhibits anchorage-independent growth by targeting ErbB4 in Ewing sarcoma [25] and impairs the proliferation of pancreatic cancer cells through suppression of KRAS [26]. In accordance with observations in solid cancer, miR-193b exhibits tumor suppressing effects in acute myeloid leukemia and T-cell acute lymphatic leukemia through suppression of c-kit and Myb, respectively [27,28]. Additionally, decreased expression of miR-193b-3p is involved in the acquisition of metastatic potential. By comparing the level of miRNA between MDA-MB-231 and its metastatic derivative, miR-193b was identified as tumor-suppressing miRNA and to suppress urokinase-type plasminogen activator (uPA) by targeting its mRNA [29]. Moreover, miR-193b-3p was able to regulate proliferation, migration, and invasion in human hepatocellular carcinoma cells by suppressing CCND1 and ETS1 [30]. Although there are many reports on the tumor-suppressing roles of miR-193b-3p, very little is known about the function of miR-193b-5p. We recently reported that miR-193b-5p competitively regulates PLK1 expression with heterogeneous nuclear ribonucleoprotein K (hnRNPK) [31]. Briefly, miR-193b-5p binds to the 3′UTR of PLK1 mRNA near the C-rich region where hnRNPK interacts and thus two trans-acting factors (i.e., miR-193b-5p and hnRNPK) competitively regulate PLK1 expression.

Several pathways have been identified to regulate the expression of miR-193b. In prostate cancer, miR-193b is an epigenetically regulated tumor suppressor [32]. It was recently reported that signal transducer and activator of transcription 5 (STAT5) regulates the expression of miR-193b, which governs hematopoietic stem and progenitor cell expansion via cytokine receptor signaling [33]. In an ovarian cancer model, microenvironmental alteration by mesothelial cells elicits the decrease in miR-193b levels, which is critical for the promotion of metastatic steps such as colonization and invasion [34]. Cystic fibrosis transmembrane conductance regulator (CFTR), which is a tumor suppressor, regulates progression of prostate cancer by suppressing uPA through miR-193b [35]. It was reported that miR-193b expression is regulated concurrently with miR-365, its cluster miRNA [36]. Their levels are commonly down-regulated in multiple myeloma, suggesting that miR-193b-365 cluster is an miRNA signature of multiple myeloma. This miR-193b-365 cluster is also involved in the control of cancer progression in epidermis squamous cell carcinoma [37]. Through *in silico* predictions and transcriptome analyses, KRAS and MAX are identified as direct targets of miR-193b and miR-365. Since miR-365 was not down-regulated in metastatic lung cancer, our study excluded the effect of miR-365 on malignant phenotypes of lung cancer cells.

It was revealed that miR-193b-3p and -5p regulate malignant phenotypes of lung cancer through the suppression of their common target genes (i.e., CCND1, AJUBA, and HEG1). Cyclin D1 encoded by the CCND1 gene is one of the frequently altered and overexpressed cell cycle regulators in cancers [38]. As an oncogene, it plays an important role in metastatic processes, including migration and invasion [39]. The cyclin D1-cyclin-dependent kinase 4 (CDK4) complex stimulates migratory and invasive abilities by triggering the phosphorylation of paxillin, which activates Ras-related C3 botulinum toxin substrate 1 (Rac1). In previous reports, we also revealed that cyclin D1 is a critical regulator of aggressiveness of lung cancer, which is responsible for brain metastasis of lung cancer cells [14,15]. Our results demonstrate that the expression of cyclin D1 is governed by two regulatory pathways. One is the miR-95-3p-elicited down-regulation. Lower miR-95-3p expression in brain metastatic lung cancer cells abrogates the orthotopic growth of lung cancer as well as inhibits the metastasis of lung cancer into the brain. The other one is the ubiquitin-specific peptidase 4 (USP4)/β-catenin pathway. USP4 is more abundant in metastatic lung cancer cells than in primary lung cancer cells, resulting in the increase in β-catenin expression through the inhibition of ubiquitin-mediated degradation [14]. Many studies have shown that cyclin D1 plays an important role in lung cancer progression. Therefore, cyclin D1 is considered to be a promising target for the treatment of lung cancer [40]. The second common target gene is AJUBA. LIM domain-containing protein Ajuba is known to be involved in various cellular processes, such as cell–cell adhesion, proliferation, and migration. In several studies, AJUBA has been identified as an oncogene with its oncogenic properties being partly due to functioning as a positive regulator of Yes-associated protein (YAP) [41–45]. AJUBA promotes the migration and invasion of esophageal squamous cell carcinoma cells (ESCC) through the up-regulation of matrix metalloproteinase 10 (MMP10) and MMP13 expression in ESCC [43]. AJUBA is highly expressed and confers cisplatin resistance on cervical cancer cells [44]. AJUBA exerts high oncogenic activity in colorectal cancer (CRC) and promotes the proliferation of CRC by inhibiting apoptotic cell death [45]. The anti-apoptotic function of AJUBA is mediated via the suppression of interferon-stimulated IFIT2, which is an apoptotic inducer. In the present study, HEG1 is also identified as one of the common target genes. HEG1, a mucin-like membrane protein is known as a specific marker for malignant mesothelioma [46]. Moreover, knockdown of the HEG1 gene dramatically decreased the survival and proliferation of mesothelioma cells. Taken together, we demonstrate that miR-193b is a new dual-strand tumor suppressing miRNA and their common target genes (i.e., CCND1, AJUBA, and HEG1) are potential targets for the treatment of lung cancer.

## Supporting information

**Supplementary Figure S1:** Search for differentially expressed miRNAs between PC14PE6 and PC14PE6/LvBr cells by microarray (**A**) Microarray analysis was performed to identify miRNA candidates. Heatmap indicate the downregulation of miRNAs in metastatic lung cancer cells (PC14PE6/LvBr4) obtained from two independent replicates. Number of Figure 1A is the fold change of miRNA expression level (Log2_Ratio_PC14PE6/LvBr4 vs PC14PE6). ‘hsa’ means that miRNAs belong to a human (Homo sapiens). (**B**) Relative levels of miR-193b-3p and -5p in microarray data were shown. Bars indicate the average of miRNA level from two independent replicates.

**Supplementary Figure S2:** Search for common target genes of miR-193b-3p and -5p The Venn diagram indicates the target gene list of miR-193b-3p and -5p as predicted by TargetScan. Common target genes including CCND1, AJUBA, and HEG1 were selected by comparison with the genes targeted by miR-193b-3p and -5p and those whose expression was decreased in metastatic PC14PE6/LvBr4 cells compared to those in parental PC14PE6 cells.

**Supplementary Figure S3:** *MMP16* is required for invasiveness of PC14PE6/LvBr4 cells, but is not a target of miR-193b-3p or -5p (**A**) Comparison of the expression level of *MMP16* between metastatic PC14PE6/LvBr4 and parental PC14PE6 cells. (**B**) PC14PE6/LvBr4 cells were transfected with *MMP16*-specific siRNA and invasiveness was determined by Transwell invasion assay. (**C**) To determine whether *MMP16* is a direct target of miR-193b-3p or -5p, the enrichment of *MMP16* mRNA in Argonaute 2-RNA immunoprecipitation (Ago2-RIP) material was assessed after overexpression of miR-193b-3p or -5p in PC14PE6/LvBr4 cells. (**D**) PC14PE6/LvBr4 cells were transfected with miRNA mimics and the expression level of *MMP16* mRNA was determined by RT-qPCR. All experiments were performed three times and data represent mean ± standard deviation (SD). Asterisk (*) denotes statistical significance of p < 0.05 calculated with the Student’s *t*-test.

**Supplementary Figure S4:** Specific siRNAs targeting CCND1, AJUBA, and HEG1 efficiently downregulate their expression PC14PE6/LvBr4 (*A-B*) and A549 (*C-D*) cells were transfected with the indicated siRNAs and the protein and mRNA expression levels of the target gene were assessed by Western blot (*A* and *C*) and RT-qPCR (*B* and *D*) analyses. All experiments were performed three times and data represent mean ± standard deviation (SD).

**Supplementary Table 1:** Verification of miRNA overexpression by miRNA mimic. After PC14PE6/LvBr4 and A549 cells were transfected with miRNA mimics, the levels of miR-193b-3p and -5p in three independent replicates were assessed by RT-qPCR using specific TaqMan primers.

## Abbreviations

3′UTR: 3′-untranslated region
Ago2-RIP: argonaute 2-RNA immunoprecipitation
AJUBA: Ajuba LIM Protein
BSA: bovine serum albumin
CCND1: Cyclin D1
CDNA: complementary DNA
CRC: colorectal cancer
DMEM: Dulbecco’s modified Eagle’s medium
ESCC: esophageal squamous cell carcinoma
FBS: fetal bovine serum
GAPDH: glyceraldehyde 3-phosphate dehydrogenase
HEG1: heart development protein with EGF like domains 1
hnRNPK: heterogeneous nuclear ribonucleoprotein K
H&E: hematoxylin and eosin
miRNA: microRNA
MMP: matrix metalloproteinase
MRE: miRNA recognition element
MT: mutated
MTDH: metadherin
pre-miRNA: precursor microRNA
RISC: RNA-induced silencing complex
RT-qPCR: reverse transcriptase quantitative polymerase chain reaction
SDS/PAGE: sodium dodecyl sulfate polyacrylamide gel electrophoresis
siRNAs: small interfering RNAs
TBST: Tris-buffered saline with Tween 20
UHRF1: ubiquitin-like with PHD and ring finger domains 1
uPA: urokinase-type plasminogen activator
USP4: ubiquitin-specific peptidase 4
WT: wild-type
