# Supplementary material for: Dual-strand tumor suppressor miR-193b-3p and -5p inhibit malignant phenotypes of lung cancer by suppressing their common targets
Source: Biosci Rep. 2019 Jul 12;39(7):BSR20190634. doi: 10.1042/BSR20190634 (PMC6630026; doi:10.1042/BSR20190634)
Supplement: Supplementary file 1 [file bsr20190634_Supp1.pptx]

## Slide 1
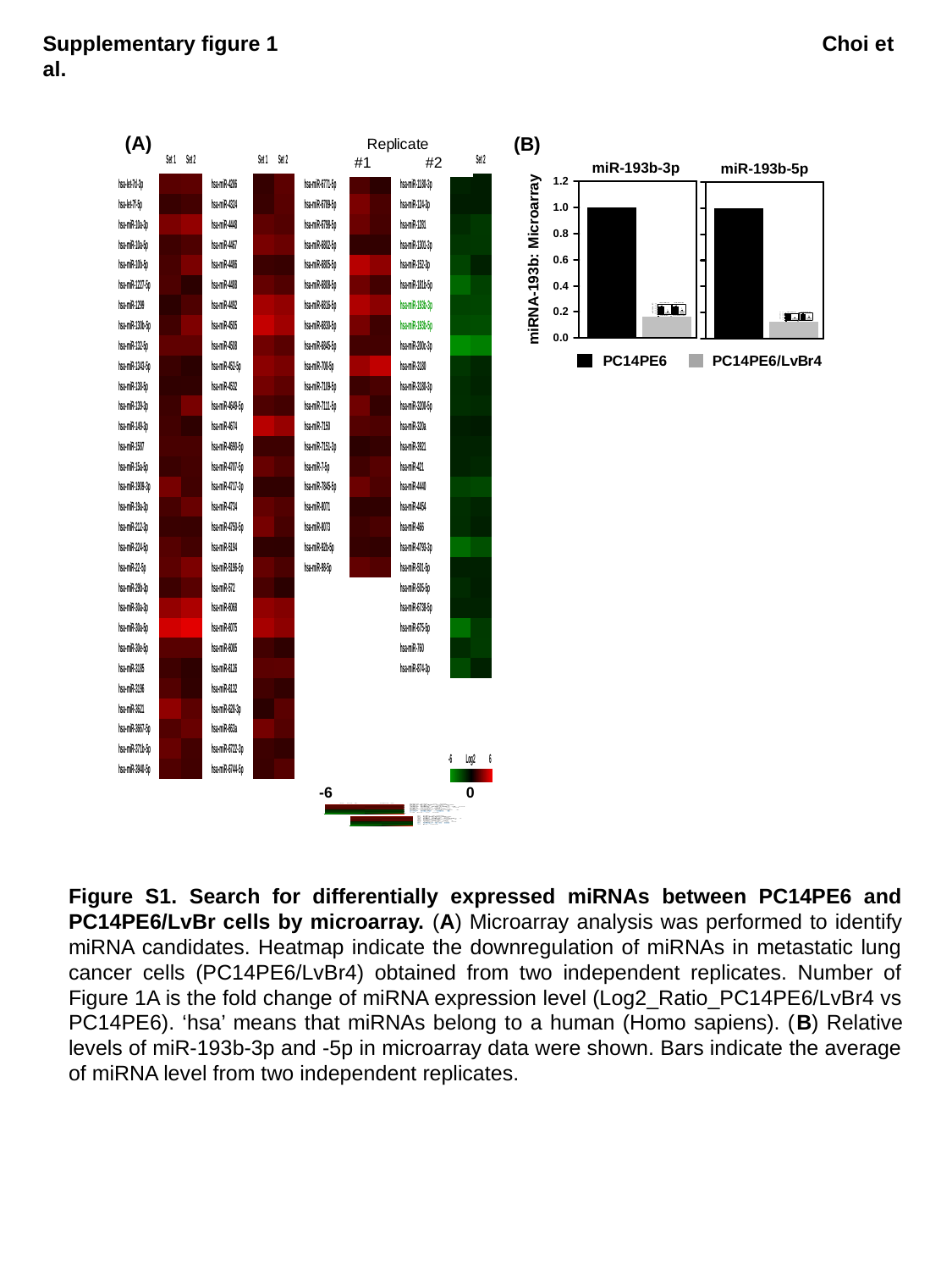

Supplementary figure 1					 Choi et al.
(A)
(B)
Figure S1. Search for differentially expressed miRNAs between PC14PE6 and PC14PE6/LvBr cells by microarray. (A) Microarray analysis was performed to identify miRNA candidates. Heatmap indicate the downregulation of miRNAs in metastatic lung cancer cells (PC14PE6/LvBr4) obtained from two independent replicates. Number of Figure 1A is the fold change of miRNA expression level (Log2_Ratio_PC14PE6/LvBr4 vs PC14PE6). ‘hsa’ means that miRNAs belong to a human (Homo sapiens). (B) Relative levels of miR-193b-3p and -5p in microarray data were shown. Bars indicate the average of miRNA level from two independent replicates.

## Slide 2
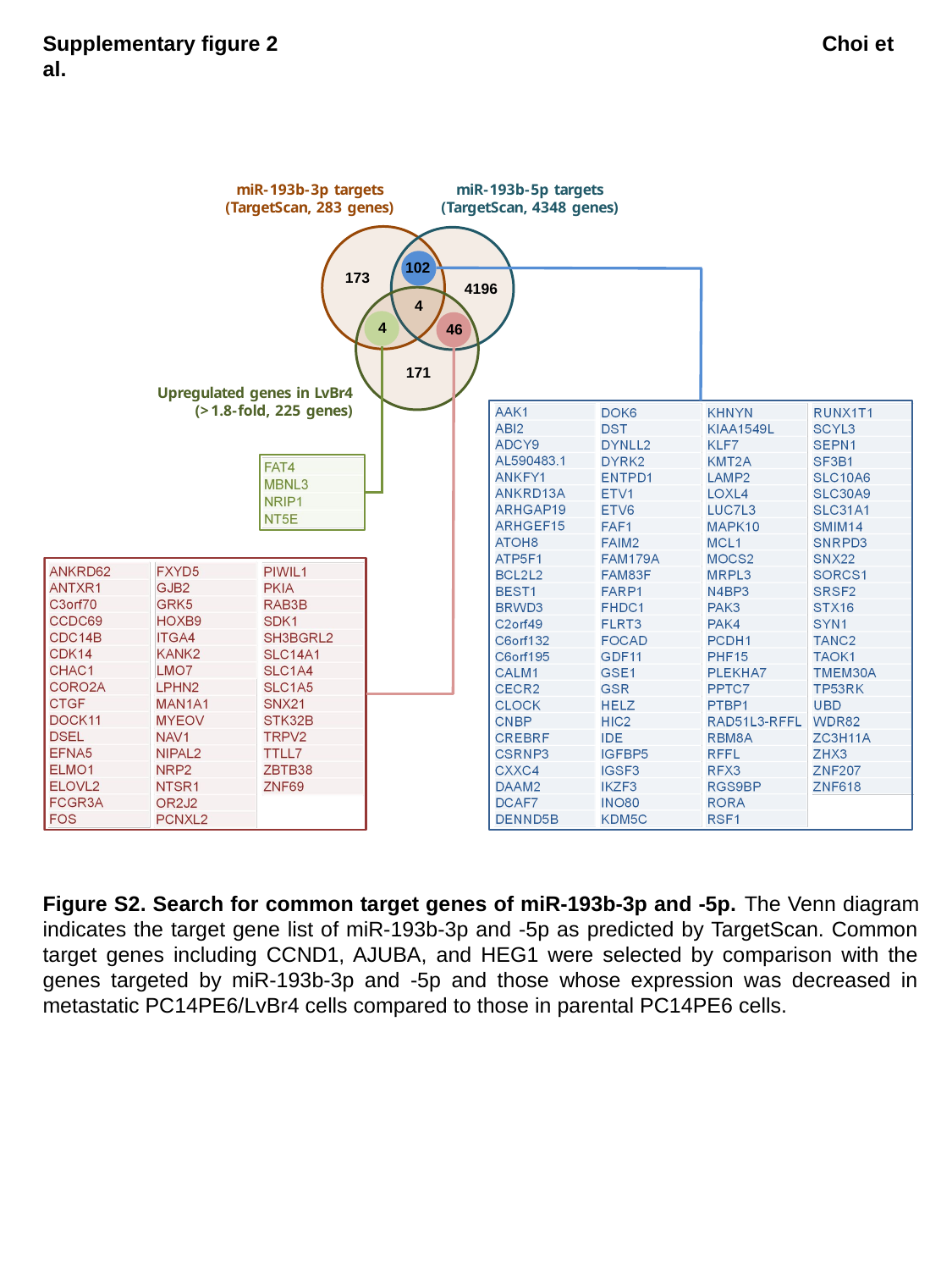

Supplementary figure 2					 Choi et al.
Figure S2. Search for common target genes of miR-193b-3p and -5p. The Venn diagram indicates the target gene list of miR-193b-3p and -5p as predicted by TargetScan. Common target genes including CCND1, AJUBA, and HEG1 were selected by comparison with the genes targeted by miR-193b-3p and -5p and those whose expression was decreased in metastatic PC14PE6/LvBr4 cells compared to those in parental PC14PE6 cells.

## Slide 3
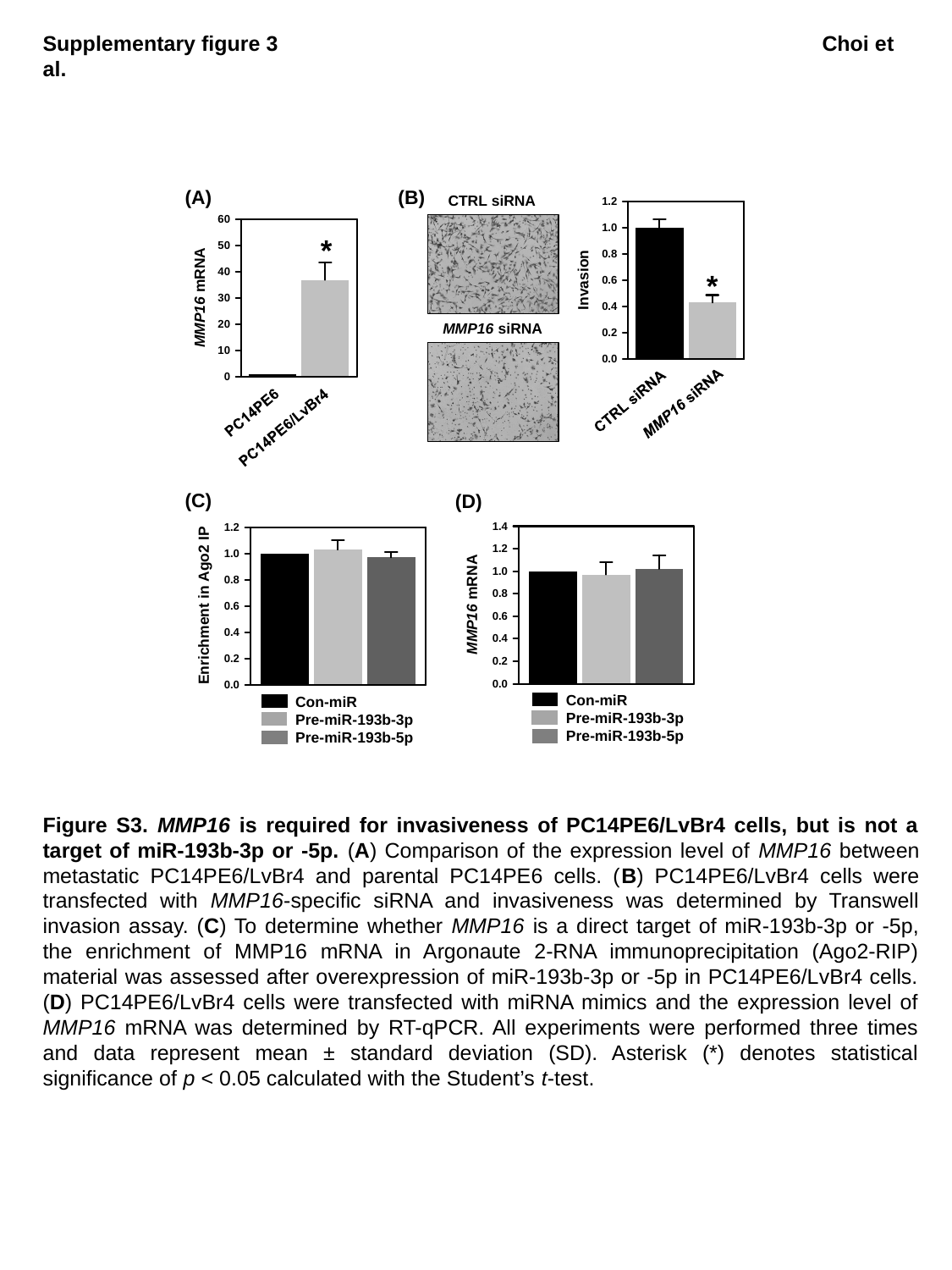

Supplementary figure 3					 Choi et al.
(A)
(B)
(C)
(D)
Figure S3. MMP16 is required for invasiveness of PC14PE6/LvBr4 cells, but is not a target of miR-193b-3p or -5p. (A) Comparison of the expression level of MMP16 between metastatic PC14PE6/LvBr4 and parental PC14PE6 cells. (B) PC14PE6/LvBr4 cells were transfected with MMP16-specific siRNA and invasiveness was determined by Transwell invasion assay. (C) To determine whether MMP16 is a direct target of miR-193b-3p or -5p, the enrichment of MMP16 mRNA in Argonaute 2-RNA immunoprecipitation (Ago2-RIP) material was assessed after overexpression of miR-193b-3p or -5p in PC14PE6/LvBr4 cells. (D) PC14PE6/LvBr4 cells were transfected with miRNA mimics and the expression level of MMP16 mRNA was determined by RT-qPCR. All experiments were performed three times and data represent mean ± standard deviation (SD). Asterisk (*) denotes statistical significance of p < 0.05 calculated with the Student’s t-test.

## Slide 4
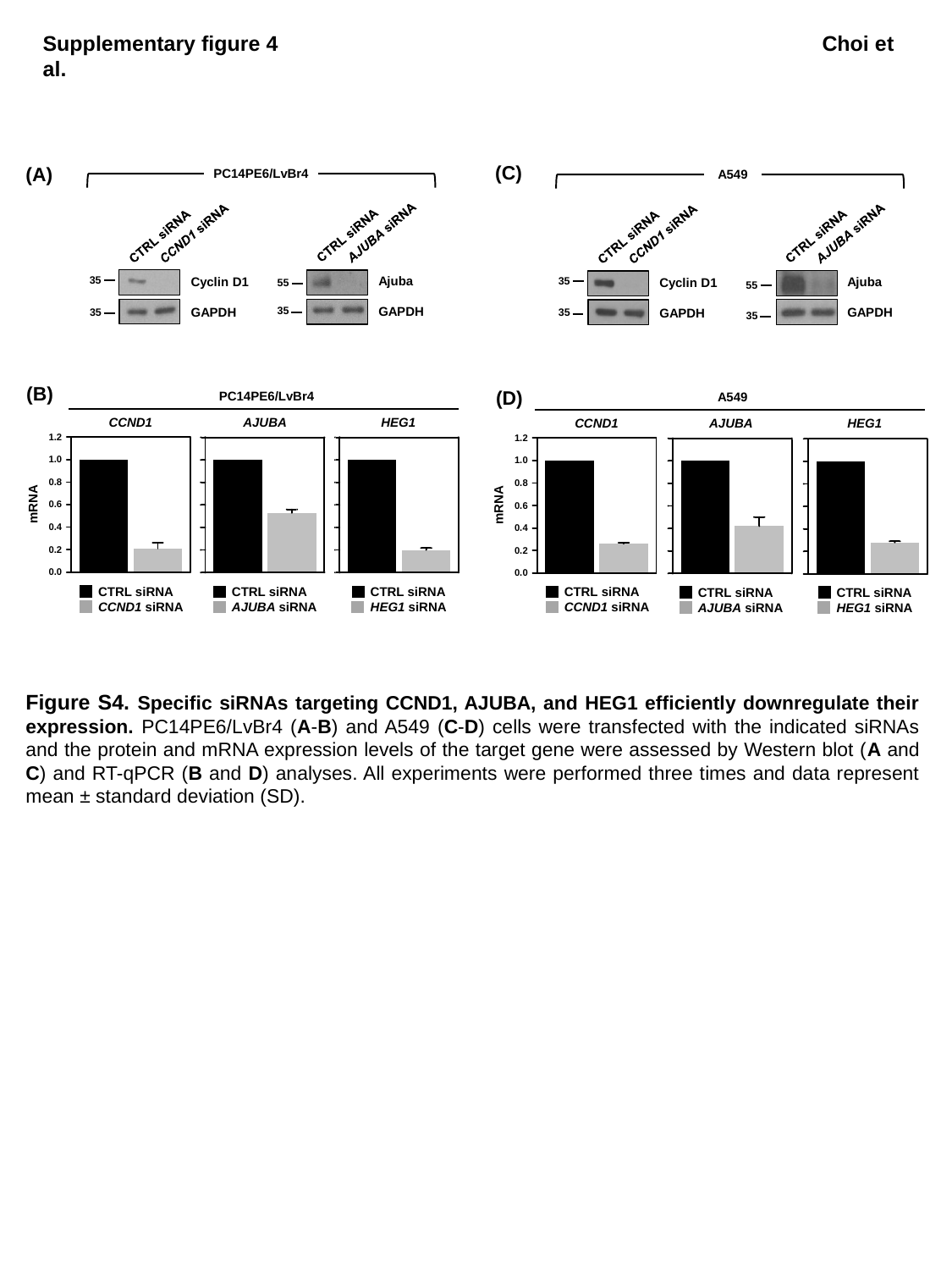

Supplementary figure 4					 Choi et al.
(C)
(A)
(B)
(D)
Figure S4. Specific siRNAs targeting CCND1, AJUBA, and HEG1 efficiently downregulate their expression. PC14PE6/LvBr4 (A-B) and A549 (C-D) cells were transfected with the indicated siRNAs and the protein and mRNA expression levels of the target gene were assessed by Western blot (A and C) and RT-qPCR (B and D) analyses. All experiments were performed three times and data represent mean ± standard deviation (SD).

## Slide 5
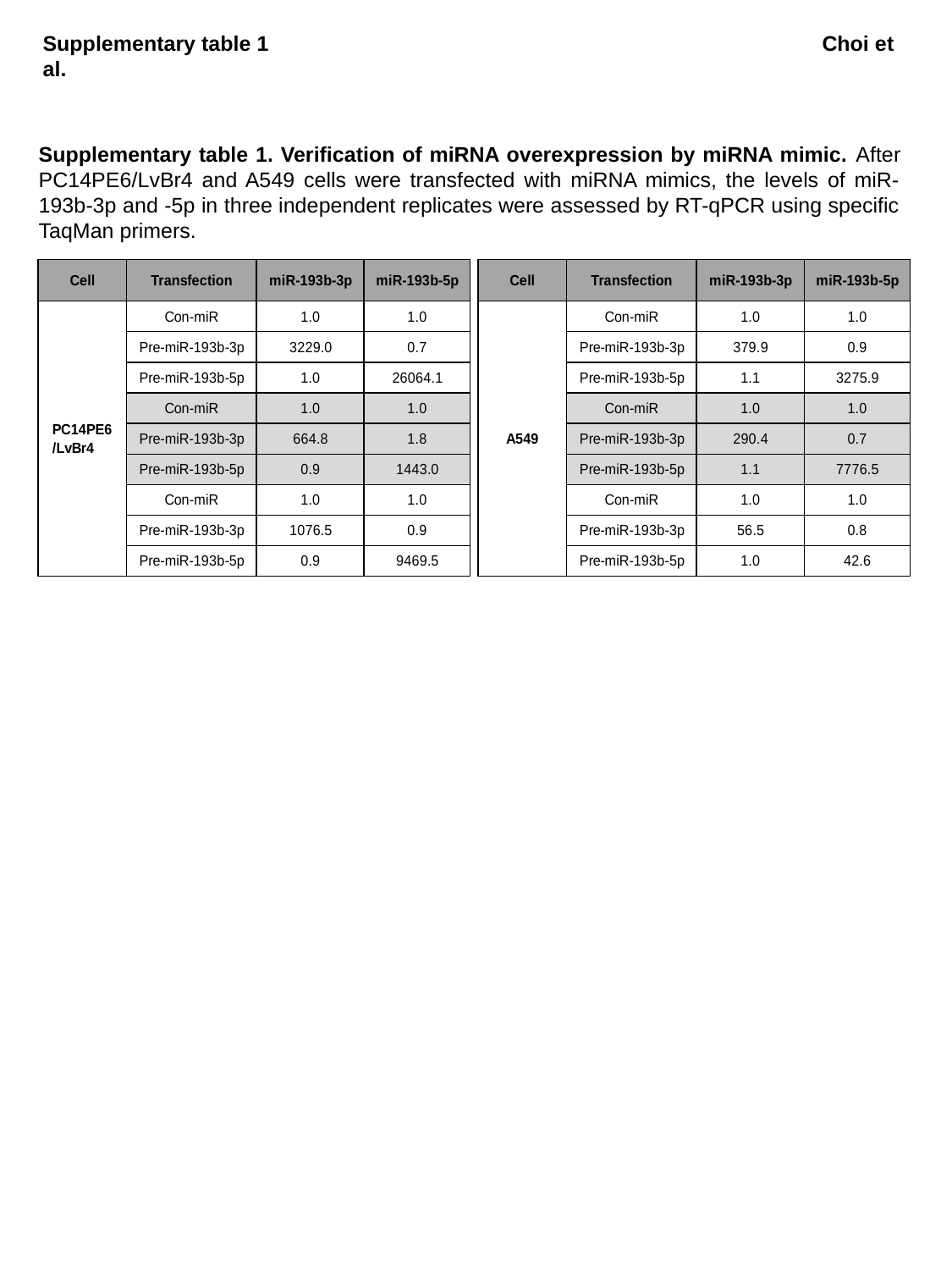

Supplementary table 1					 Choi et al.
Supplementary table 1. Verification of miRNA overexpression by miRNA mimic. After PC14PE6/LvBr4 and A549 cells were transfected with miRNA mimics, the levels of miR-193b-3p and -5p in three independent replicates were assessed by RT-qPCR using specific TaqMan primers.
